# Subduing the Inflammatory Cytokine Storm

**DOI:** 10.3390/ijms252011194

**Published:** 2024-10-18

**Authors:** Raymond Kaempfer

**Affiliations:** Department of Biochemistry and Molecular Biology, Faculty of Medicine, The Hebrew University of Jerusalem, Jerusalem 9112102, Israel; kaempfer@hebrew.edu

**Keywords:** cytokine storm, inflammatory cytokines, lethal toxic shock, immune costimulation, costimulatory receptor, B7, CD28, homodimer interface, mimetic peptides

## Abstract

The inflammatory cytokine response is essential for protective immunity, yet bacterial and viral pathogens often elicit an exaggerated response (“cytokine storm”) harmful to the host that can cause multi-organ damage and lethality. Much has been published recently on the cytokine storm within the context of the coronavirus pandemic, yet bacterial sepsis, severe wound infections and toxic shock provide other prominent examples. The problem of the cytokine storm is compounded by the increasing incidence of multidrug-resistant bacterial strains. We created an incisive molecular tool for analyzing the role of the B7/CD28 costimulatory axis in the human inflammatory response. To attenuate the cytokine storm underlying infection pathology, yet preserve host defenses, we uniquely targeted the engagement of CD28 with its B7 co-ligands by means of short peptide mimetics of the human CD28 and B7 receptor homodimer interfaces. These peptides are not only effective tools for dissecting mechanism but also serve to attenuate the inflammatory response as a broad host-oriented therapeutic strategy against the cytokine storm. Indeed, such peptides protect mice from lethal Gram-positive bacterial superantigen-induced toxic shock even when dosed in molar amounts well below that of the superantigen and show promise in protecting humans from the severe inflammatory disease necrotizing soft tissue infections (‘flesh-eating’ bacterial sepsis) following traumatic wound injuries.

## 1. Introduction

The centuries-old quote “Too much of a good thing can be a bad thing” certainly applies to the inflammatory response. Whereas the inflammatory cytokine response is essential for eliciting protective immunity, pathogens that include bacteria, viruses and toxins often elicit a vastly exaggerated response, termed a “cytokine storm”, that is harmful to the host and may cause multi-organ damage and lethality. A cytokine storm and cytokine release syndrome are life-threatening systemic inflammatory syndromes involving elevated levels of circulating cytokines and immune cell hyperactivation [1,2,3]. Here, we review how the initial need to develop biodefense against lethal toxic shock induced by Gram-positive bacterial superantigen toxins, feared as biological weapons as they are deadly yet not infectious, led to the identification of the costimulatory receptors CD28 and B7 (CD80 and CD86) as novel superantigen receptors and as targets for subduing a cytokine storm and to the design of short peptide mimetics of the self-adhesive homodimer interfaces within the protein structures of these three receptors. These mimetic peptides enabled resolution of the molecular mechanism of the elicitation of a cytokine storm, and led to the discovery that they are capable of attenuating the formation of the B7/CD28 costimulatory axis, and thereby achieve attenuation of a cytokine storm, also seen in other pathologies. Such peptides show promise more broadly in protecting humans from the severe inflammatory disease, necrotizing soft tissue infections, and from polymicrobial sepsis.

## 2. Results

A major breakthrough in our understanding of how to block a lethal inflammatory cytokine storm came from the need to develop a biodefense against bacterial superantigen toxins. Superantigens are among the most lethal of toxins. Superantigens produced by *Staphylococcus aureus* and *Streptococcus pyogenes* trigger a vastly excessive cellular immune response that can lead to lethal toxic shock [4,5,6]. Our early studies of the molecular biology of human cytokine gene expression, some even before cDNA cloning [7,8,9] and its disturbance in disease [10,11,12,13], prompted the Pentagon to seek our involvement in developing a defense against this potential biological weapon. That led us to the discovery of the vital and broad role of the axis formed between the B7 and CD28 immune costimulatory receptors in causing a cytokine storm and the broad power of subduing a harmful cytokine storm by attenuating the formation of this costimulatory axis with short mimetic peptides.

### 2.1. Superantigens Induce a Lethal Cytokine Storm by Directly Engaging B7 and CD28

In a conventional immune response, 0.01% of T cells interact with antigens to orchestrate a limited and highly specific immune response, whereas superantigens engage 20% to 30% of T cells, regardless of antigen specificity, leading to polyclonal expansion and release of proinflammatory cytokines. This excessive activation of the host response can result in septic shock and multiple organ failure [4,5,6,14]. For many years, it was known that bacterial superantigens induce a cytokine storm by binding outside the cell directly to the major histocompatibility class II (MHC-II) molecule on the antigen-presenting cell (APC) and to the T-cell receptor (CD3) [14]. Bypassing the restricted presentation of conventional antigens, superantigens bind directly to most major histocompatibility (MHC) class II molecules and stimulate almost all T cells bearing particular domains in the variable portion of the β chain (Vβ) of the T-cell receptor, without need for processing by antigen-presenting cells [14]. The T-cell receptor interacts with superantigens through the outer face of its Vβ domain, a region not involved in ordinary antigen recognition [14]. We discovered that in addition to MHC-II molecules and the T-cell receptor, not only the costimulatory receptor CD28 but also its co-ligands B7-1 (CD80) and B7-2 (CD86) function as direct and critical receptors for superantigen toxins. This surprising discovery enabled the design of entirely novel antagonist peptides.

**Figure 1:**
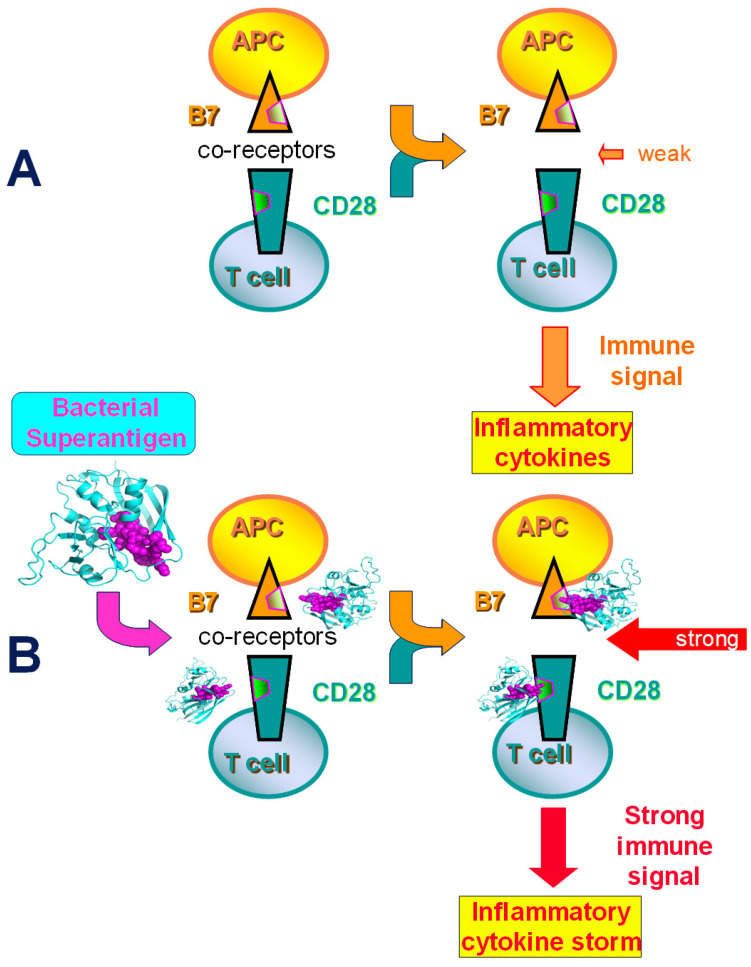
To evoke a cytokine storm, superantigen toxins must bind onto CD28 and B7 costimulatory receptor homodimer interfaces. (**A**) B7/CD28 receptor engagement is a primary immune checkpoint, essential for effective T cell activation. B7 expressed on the antigen-presenting cell (APC) engages CD28 on the T cell (arrow colors denote receptor engagement). This relatively weak interaction elicits a moderate immune signal in the T cell to induce inflammatory cytokines (orange arrows). (**B**) Bacterial superantigens induce a lethal cytokine storm by directly engaging, through their structurally conserved β-strand–hinge–α-helix domain (purple), both CD28 and B7 co-receptors at their receptor homodimer interfaces (yellow and green trapezoids) (purple arrow). Binding of a superantigen to these costimulatory receptors evokes a strong B7/CD28 interaction, thereby eliciting a powerful immune signal into the T cell that results in the induction of an inflammatory cytokine storm (red arrows) [14,15,16,17].

As Figure 1A shows, under normal circumstances, B7 receptors expressed on the APC and CD28 expressed on the T cell interact with weak to moderate affinity to provoke an adequate immune signal in the T cell for the induction of inflammatory cytokines that underlies the generation of protective immunity. Induction of human T helper 1 (Th1) cytokine gene expression by divergent staphylococcal and streptococcal superantigens, we found, is inhibited by a dodecamer peptide that protects mice from their lethal effect [14]. This peptide is a mimetic of the β-strand(8)/hinge/α-helix(4) domain in staphylococcal enterotoxin B (SEB), structurally conserved among the staphylococcal and streptococcal superantigens, yet remote from their MHC-II and T-cell receptor binding sites, being located on the opposite side in the superantigen protein structure [14,15]. This dodecamer peptide not only protects mice from lethal challenge with various staphylococcal and streptococcal superantigens but also rescues mice from death when administered several hours after the toxin [14].

The protected mice rapidly developed immunity to lethal shock [14]. Mice that had survived a lethal challenge with SEB due to protection by the dodecamer peptide were re-challenged two weeks later with the same dose of SEB, this time in the absence of peptide antagonist, and survived; similar results demonstrating protection from a cytokine storm by the peptide were obtained with streptococcal pyrogenic exotoxin A (SPEA) [14]. Indeed, mice protected by this antagonist peptide rapidly developed broad-spectrum immunity to lethal shock [14]. These mice rapidly acquired cross-protection by protective antibodies. Mice that were protected from SEB-induced lethal shock by a single dose of dodecamer peptide survived in the absence of any further antagonist peptide administration and with subsequent challenge by the double dose of SEB, followed by challenge with a lethal dose of SPEA, followed by challenge with a double dose of SPEA, and followed by a lethal dose of toxic shock syndrome toxin-1 (TSST-1) [14]. Adoptive transfer of serum from these surviving mice was sufficient to protect naive mice against lethal challenge with SEB [14]. Rapidly evolving resistance to toxin challenge is thus based on the generation of protective antibodies in mice surviving superantigen toxin exposure, owing to the antagonist peptide. These results showed that while the deleterious cytokine storm induced by the pathogen was attenuated, the protective immune response remained fully intact.

Because the newly identified β-strand(8)/hinge/α-helix(4) domain is essential for superantigen lethal toxicity, as demonstrated by the superantigen mimetic peptide [14], we considered that beyond the MHC-II molecule and T-cell receptor, it may engage a third receptor essential for toxicity and a cytokine storm. We next discovered that the bacterial superantigen uses its highly conserved β-strand–hinge–α-helix domain [14,15] to strongly enhance CD28 signaling by directly engaging the homodimer interfaces of CD28 [15] as well as of B7-2 [16] and B7-1 [18] (Figure 1B). Hitherto, CD28 was not known to bind microbial components. It became clear that superantigens co-opt CD28 as their receptor, and that to induce a cytokine storm, superantigens must bind directly into the dimer interface of CD28 [15]. Blocking access of a superantigen to CD28 is sufficient to block lethal toxic shock [15]. Peptide mimetics of the CD28 dimer interface inhibited the induction of Th1 cytokines and protected mice from lethal superantigen exposure [15].

Mutation of the β-strand(8)/hinge/α-helix(4) superantigen domain abolished inflammatory cytokine gene induction and lethality [15]. Structural analysis showed that when a superantigen binds to the T-cell receptor on the T cell and MHC class II molecule on the APC, CD28 can be accommodated readily as a third superantigen receptor in the quaternary complex, with the CD28 dimer interface oriented towards the β-strand(8)/hinge/α-helix(4) domain in the superantigen [15].

### 2.2. Superantigen Toxins Trigger B7/CD28 Costimulatory Receptor Engagement

Binding of the superantigen triggers intercellular B7/CD28 costimulatory receptor engagement [16]. By strongly enhancing both the B7-2/CD28 costimulatory receptor interaction [16], as well as the tighter B7-1/CD28 interaction [17,18], staphylococcal and streptococcal superantigens hyper-induce inflammatory cytokines, leading to a cytokine storm (Figure 1B). The finding that CD28 and its two B7 co-ligands are critical receptors for the superantigen toxins broadened the scope of microbial pathogen recognition mechanisms and provided a novel approach for designing therapeutics that protect against lethal toxic shock.

### 2.3. Design of Peptide Mimetics of the Human CD28 and B7 Receptor Homodimer Interfaces

Once it was shown that the superantigen SEB binds directly to CD28 [15], we mapped the binding site in CD28 to its composite homodimer interface [15]. One of the first homodimer interface mimetic peptides we designed, octapeptide p*2TA* [15], inhibits the induction of interleukin-2 (IL-2), interferon-γ (IFN-γ), as well as tumor necrosis factor-α (TNF-α) in primary human peripheral blood mononuclear cells (PBMCs), by widely diverse staphylococcal superantigen toxins: SEB, SEA, and TSST-1 [15]. Whereas SEB engages only the MHC-II α-chain, SEA also binds the β-chain [15]; TSST-1 is only 6% homologous with SEB and binds the T-cell receptor via a different domain [19]. p*2TA* binds and antagonizes superantigens and protects mice from lethal toxin challenge with SEB [15].

**Figure 2:**
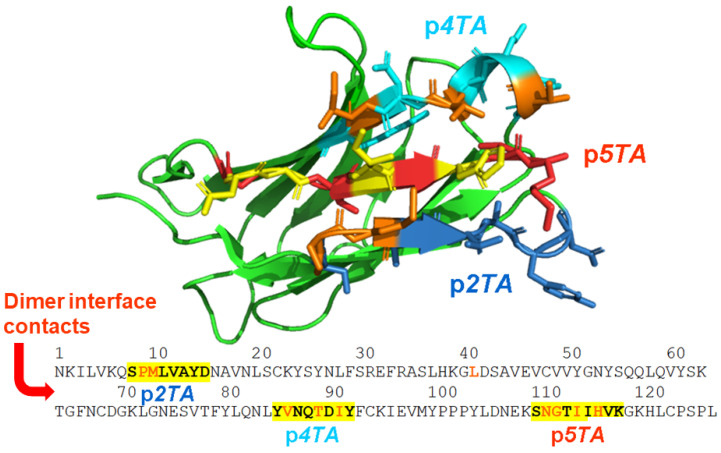
Mimetic peptide p*2TA*, p*5TA*, and p*4TA* domains at the homodimer interface of costimulatory receptor CD28. In the CD28 extracellular domain sequence, peptide sequences are highlighted in yellow, with dimer interface contact residues in red. In the cartoon model of the extracellular domain of CD28 (green; 1yjd.pdb), a single beta-barrel, p*2TA* is shown in sticks in dark blue with 2 dimer interface contacts in orange [15], p*5TA* in red with 4 dimer interface contacts in yellow, and p*4TA* in cyan with 3 dimer interface contacts in orange [18].

Figure 2 illustrates the dimer interface contacts within the first-generation mimetic p*2TA* domain and its location within the CD28 dimer interface. Subsequently, second-generation mimetic peptides p*4TA* and p*5TA* that harbor more and distinct dimer interface contacts [18] were created.

**Figure 3:**
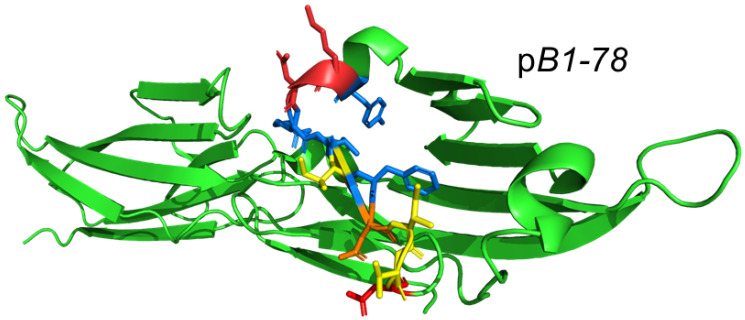
Design of B7-1 (CD80) homodimer interface mimetic peptide. In the cartoon model of the B7-1 extracellular domain (green; 1dr9.pdb), a double β-barrel and amino acid residues forming p*B1-78* (YKNRTIFDITN) are modeled in sticks, with 4 residues (underlined) that make the homodimer interface contacts shown in yellow and orange [18].

Figure 3 illustrates the location of B7-1 (CD80) homodimer interface mimetic peptide p*B1-78* within the B7-1 homodimer interface [18]. Like the CD28 mimetic peptides, B7-1 and B7-2 dimer interface mimetic peptides each attenuated the induction of IL-2, IFN-γ and TNF-α in human PBMCs by SEB or by αCD28 monoclonal antibody jointly with αCD3 [16,18], which is a model for conventional T cell activation [15,20].

Unlike CD28 and B7-1, which form covalent homodimers, B7-2 (CD86) forms only an extremely weak, noncovalent homodimer and exists mostly as a monomer on the cell surface, which renders the binding of superantigens into the B7-2 dimer interface even more remarkable [16]. Indeed, short peptide mimetics of the B7-2 dimer interface are superantigen antagonists that downregulate the induction of IL-2, IFN-γ, and TNF-α by SEB and protect mice from lethal superantigen toxic shock, whereas B7-2 peptide mimetics derived from domains outside the dimer interface fail to do so [16].

### 2.4. The Homodimer Interface Mimetic Peptides Attenuate Intercellular B7/CD28 Engagement

Within the folded extracellular domains of CD28, as well as of B7-1 and B7-2, the homodimer interface is located far from the site where the co-ligand binds [15,16,18,21,22,23,24]. Using surface plasmon resonance, we could show that the mimetic peptide p*2TA*, derived from the self-adhesive CD28 dimer interface, binds back directly into the dimer interface of CD28 with moderate, low micromolar affinity [18]. When CD28 was expressed on the surface of HEK293T cells by transfection, p*2TA* inhibited binding of soluble B7-2 to cell-surface CD28 [18]. Conversely, when B7-2 was expressed on the surface of HEK293T cells by transfection, p*2TA* strongly inhibited the binding of soluble CD28 to cell-surface B7-2 [18]. This strategy allowed for the study of the B7-2/CD28 interaction, in the absence of multiple ligand–receptor interactions that underlie synapse formation between APC and T cells, involving not only MHC class II/T-cell receptor interaction but also additional costimulatory ligand pairs that could mask the contribution of B7-2/CD28 engagement [16]. Use of HEK293T cells avoids additional interactions that occur between APC and T cells, placing the focus on the B7-2/CD28 interaction [18].

Using flow cytometry, it was shown that p*2TA* attenuates intercellular B7-2/CD28 engagement [18]. Moreover, p*2TA* attenuated intercellular B7-1/CD28 engagement, which was not only more extensive but also more resistant to inhibition by p*2TA* than B7-2/CD28 engagement [18], reflecting the significantly higher affinity of B7-1 for CD28 [25]. Likewise, CD28 mimetic peptides p*4TA* and p*5TA* attenuated both intercellular B7-1/CD28 and B7-2/CD28 engagement [18]. Remarkably, B7-1 and B7-2 dimer interface mimetic peptides also attenuated the intercellular engagement of CD28 yet showed tight selectivity for the cognate B7 receptor [18]. This selectivity reinforces the concept that the costimulatory receptor dimer interface mimetic peptides bind back into the self-adhesive dimer interface that they are derived from and thereby regulate the ligand interactions of the cognate receptor [18]. B7-1 and B7-2 dimer interface mimetic peptides each diminish signaling through CD28 for inflammatory cytokine production [18].

Hence, the interaction between CD28 and its two B7 co-ligands can be attenuated through the CD28 and B7 dimer interfaces. Formation of the B7/CD28 costimulatory axis is controlled through the B7 and CD28 receptor homodimer interfaces [18]. These findings demonstrate the protective potential against a cytokine storm of attenuating proinflammatory signaling via these protein domains using mimetic peptides. Down-regulation of IL-2 induction, a cytokine specific for T cells, shows that the peptide mimetics attenuate proinflammatory signaling downstream of CD28 [18].

### 2.5. Dimer Interface Mimetic Peptides Protect Mice from Lethal Toxic Shock in Doses Far Sub-Molar to the Superantigen

By binding into the CD28 [15] and B7 [16] homodimer interfaces through a conserved 12-amino-acid β-strand–hinge–α-helix domain remote from their MHC class II and T-cell receptor binding sites [14], bacterial superantigens strongly enhance intercellular synapse formation mediated by the interaction of cell-surface CD28 with B7-2, as well as with B7-1 co-ligand [16,17], thereby eliciting a hyperinflammatory response (Figure 1B). CD28 and B7-2 dimer interface mimetic peptides compete with the cell-surface receptors in binding to the superantigen, thereby inhibiting access of the superantigen to its CD28 and B7-2 targets and protecting from lethal toxic shock [15,16].

**Figure 4:**
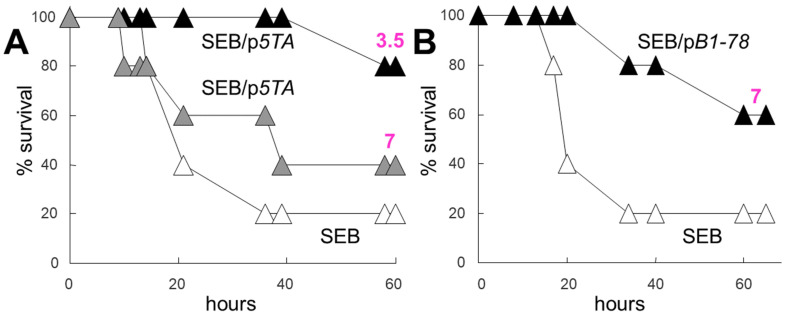
Dosed at an amount far sub-molar to the toxin, CD28 and B7-1 dimer interface mimetic peptides protect mice from lethal superantigen challenge. Peptides were injected i.p. 30 min before challenge. (**A**) Mice (*n* = 5 per group) were injected with 7.5 µg SEB alone or together with 0.045 µg p*5TA* (grey symbols) or 0.09 µg p*5TA* (black symbols). (**B**) Mice (*n* = 5 per group) were injected with 7.5 µg SEB alone or together with 0.052 µg p*B1-78* (black symbols). The molar ratio SEB/peptide is shown in the color purple (reproduced after [18]).

The induction of an inflammatory cytokine response in human PBMCs, whether caused by αCD3/αCD28 or by SEB, was sensitive to inhibition by the dimer interface mimetic peptides that attenuate the formation of the B7/CD28 costimulatory axis, suggesting that these peptides might be capable of protecting mice from lethal superantigen challenge, even when dosed in molar amounts well below that of the superantigen, to exclude the role of peptide competition with superantigen in binding to B7 or CD28. As Figure 4 shows, SEB induced pronounced mortality within hours, yet when a low dose of p*5TA* was administered at the time of exposure to SEB, the CD28 mimetic peptide was able to provide protection from death (Figure 4A). Mice that received p*5TA* at a dose 7-fold lower than that of SEB, in terms of molar amount (Figure 4A, grey symbols), showed marked survival benefit [18]. Mice that received p*5TA* at a dose 3.5-fold lower than that of SEB, in terms of molar amount, showed near-complete survival (Figure 4A, black symbols) [18]. Thus, protection by peptide is dose-dependent. Likewise, mice that received p*B1-78* at a dose 7-fold sub-molar to the SEB dose showed marked survival (Figure 4B) [18].

The explanation of these in vivo results is that at sub-molar doses, the dimer interface mimetic peptides protect not through competition with CD28 and B7-1 for the superantigen but by attenuating signaling through CD28 via an inhibition of B7/CD28 costimulatory axis formation, which is essential for eliciting lethality via a cytokine storm [18].

### 2.6. Subduing the Cytokine Storm: Beyond Superantigen Lethal Shock

The first-generation mimetic peptide p*2TA*, derived from the CD28 homodimer interface (Figure 2), proved effective as an antagonist of CD28 signaling not only against superantigen toxicity but also against Gram-positive as well as Gram-negative bacterial infections and polymicrobial sepsis, with a clear therapeutic benefit. Thus, p*2TA* attenuates toxic shock and necrotizing soft tissue infection induced by *Streptococcus pyogenes* [26]. p*2TA* acts as a preventive and therapeutic agent in mouse models of severe bacterial sepsis and Gram-negative bacterial peritonitis [27].

The peptide blocked inflammatory cytokine induction in human PBMCs by Gram-negative bacterial lipopolysaccharide (LPS) and improved the survival of mice after lethal LPS challenge. LPS is a potent adjuvant of the T-helper type 1 (Th1) response [27]. p*2TA* protects against lethal *Escherichia coli* O18:K1 peritonitis [27].

A single dose of p*2TA* improved survival of mice after cecal ligation/puncture (CLP) and increased bacterial clearance following CLP from the site of infection in the peritoneum and blood, as well as systemically in other organs enriched with macrophages [27]. Moreover, p*2TA* limited neutrophil influx after CLP in the spleen, liver, and kidneys; the peptide also reduced levels of KC, a murine chemokine homologue of human IL-8, a potent neutrophil chemoattractant, in the spleen and liver [27].

Radiation enteritis results from an excessive inflammatory cytokine response induced upon total-body irradiation that is used to combat cancer. The radiation kills the cancer cells, and cellular components released from the dead cells induce an autoimmune response that damages the intestinal tract especially [28]. A single administration of p*2TA* prevented inflammatory and thrombotic reactions in total-body irradiated mice and protected against gastrointestinal injury [28]. Hence, attenuation of CD28 signaling is a promising therapeutic approach for the mitigation of radiation-induced tissue injury [28].

Thus, the CD28 homodimer interface mimetic peptide p*2TA* does not target the pathogen but rather acts to modulate the host immune response, rendering its activity not pathogen-specific.

**Figure 5:**
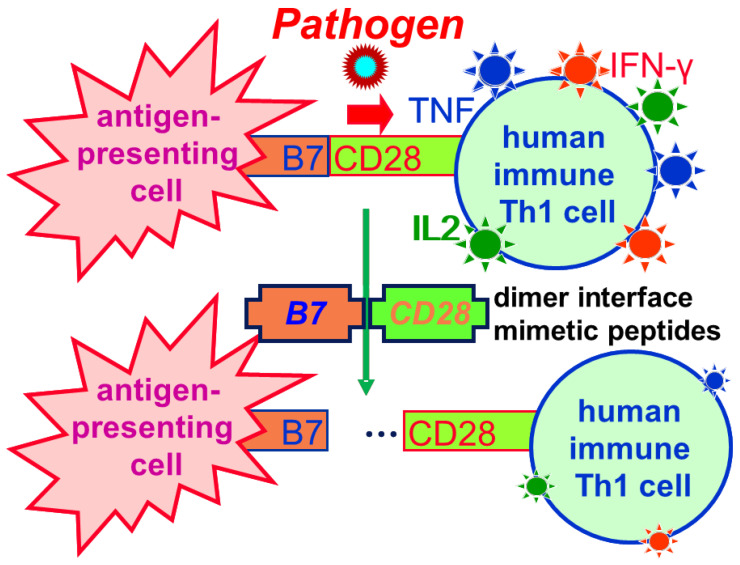
Inflammatory cytokine storm: the killer. (**Top**) Through the B7/CD28 costimulatory axis, pathogens hyperactivate Th1 cells to evoke a cytokine storm (strong red arrow). (**Bottom**) Host-oriented therapeutic B7 and CD28 homodimer interface mimetic peptides attenuate B7/CD28 receptor engagement (thin green arrow) and thereby downregulate the human inflammatory Th1 cytokine response to control and prevent a cytokine storm, a strategy unlikely to be overcome by pathogen mutation. Importantly, the mimetic peptides leave a basal cytokine response intact.

Figure 5 illustrates how by attenuating formation of the B7/CD28 costimulatory axis, B7 and CD28 homodimer interface mimetic peptides reduce the host inflammatory cytokine storm induced by various pathogens. Notably, although T cell activation is triggered by TCR/MHC-II binding, TCR/MHC-II engagement alone cannot compensate for the reduction in costimulatory B7/CD28 signaling that results from the binding of mimetic peptides. These peptides, therefore, may be used to treat infections from various sources having a substantial inflammatory component.

### 2.7. Clinical Application of CD28 Dimer Interface Mimetic Peptide

As a first-generation peptide mimetic of CD28, p*2TA* proved less potent as an inhibitor of inflammatory cytokine induction in human PBMCs and as a superantigen antagonist compared to mimetic peptides that were developed subsequently, as reviewed above, yet because of its early design, p*2TA* also became the first candidate for clinical trials. The severe inflammatory disease, necrotizing soft-tissue infection (NSTI) (‘flesh-eating’ bacterial sepsis), which occurs following traumatic wound injuries, is caused by a broad spectrum of bacteria, often polymicrobial and multidrug-resistant. NSTI results in tissue necrosis and systemic signs of sepsis. No drug is available today against severe sepsis. NSTI cases have high morbidity and mortality rates despite aggressive surgical debridement and antibiotic therapy [29]. In clinical trials using human patients with NSTIs, a single administration of p*2TA* (named here AB103 and Reltecimod) significantly enhanced the resolution of organ dysfunction and failure, attenuated an inflammatory cytokine storm, and showed excellent safety [29,30].

## 3. Discussion

The CD28 and B7 dimer interface mimetic peptides described above proved not only to be effective tools for dissecting molecular mechanism but also serve to attenuate the inflammatory response as a host-oriented therapeutic strategy against a cytokine storm. This may well have broad implications for the treatment of various diseases that cause pathology by provoking a cytokine storm or cytokine release syndrome, including chimeric antigen receptor T cell therapy of cancer, e.g., [31]. By attenuating yet not eliminating inflammatory signaling through a moderate affinity for B7 and CD28, the mimetic peptides provide a selective approach that, while preventing a cytokine storm, leaves a basal cytokine response intact to allow for a return to immune homeostasis with no compromise of host defenses, which is essential for enabling elimination of the pathogen [18].

The mimetic peptides of superantigen CD28 and B7 mitigate a harmful cytokine storm yet do not induce T cell anergy. Mice protected by a superantigen mimetic peptide from superantigen toxicity rapidly developed broad-spectrum immunity from lethal shock induced by the same or different superantigens [14]. As detailed in Section 2.6 and Section 2.7 above, CD28 mimetic peptide p*2TA* protects mice from a wide variety of lethal infections and showed excellent safety in humans while protecting them from severe sepsis. Thus, the dimer interface mimetic peptides do not induce immune deficiency in vivo.

Attempts have been made to create small-molecule inhibitors of B7/CD28 engagement through phage display and other protein engineering methods, e.g., [32,33,34,35]. Such small molecule studies did not include testing for protection from a lethal cytokine storm and go beyond the scope of this review.

The host-oriented therapeutic strategy against a cytokine storm reviewed here is unlikely to allow for the development of pathogen resistance. This is especially important in this era, where the incidence of multidrug-resistant strains is increasing.

## Data Availability

Not applicable.
